# RAPD PCR detects co-colonisation of multiple group B streptococcus genotypes: A practical molecular technique for screening multiple colonies

**DOI:** 10.1016/j.mimet.2021.106322

**Published:** 2021-11

**Authors:** Ka-Ning To, Oliver Powell, Dorota Jamrozy, Rachel Kopunova, Kyriaki Anastasiadou, Amadou Faal, Ousman Secka, Victoria Chalker, Kirsty Le Doare, Elita Jauneikaite

**Affiliations:** a Department of Infectious Disease, Imperial College London, Norfolk Place, London W2 1PG, UK; b Institute of Infection and Immunity, St George's University of London, Cranmer Terrace, London SW17 0RE, UK; c MRC Centre for Molecular Bacteriology and Infection, Department of Infectious Disease, Imperial College London, 14 Armstrong Road, London SW7 2DD, UK; d Wellcome Sanger Institute, Hinxton, Cambridge CB10 1SA, UK; e Department of Medicine, Imperial College London, Norfolk Place, London W2 1PG, UK; f Department of Infectious Disease Epidemiology, School of Public Health, Imperial College London, Norfolk Place, London W2 1PG, UK; g Microbiology Laboratories, MRC Unit The Gambia at LSHTM, Fajara, P. O. Box 273, The Gambia; h Respiratory and Vaccine Preventable Bacteria Reference Unit, Public Health England, 61 Colindale, London NW9 5EQ, UK; i NHIR Health Protection Research Unit in Healthcare Associated Infections and Antimicrobial Resistance, Department of Infectious Disease, Imperial College London, Hammersmith Campus, Commonwealth Building, London, W12 0NN, UK

**Keywords:** Group B *Streptococcus*, *S. agalactiae*, RAPD PCR, Screening, Co-colonisation, Genetic diversity, Multiple serotypes, GBS, group B s*treptococcus*, MLST, multilocus sequence typing, RAPD, random amplification of polymorphic DNA, SNP, single nucleotide polymorphism, WGS, whole genome sequencing

## Abstract

Group B *Streptococcus* (GBS) is a leading cause of neonatal meningitis, pneumonia, and sepsis. The biggest contributing factor of neonatal infections is due to vertical transmission from maternal colonisation of GBS in the genitourinary tract. Multiple serotype colonisation is often not investigated in epidemiological studies, but it is an important consideration for serotype-based vaccine development and implementation to ensure less abundant serotypes are not under-represented. In this study, we show that RAPD PCR is a quick tool useful in screening the presence of genetically different strains using multiple colony picks from a single patient swab. We observed a maximum of five different GBS strains colonising a single patient at a specific time.

## Highlights

• Co-colonisation of GBS strains is present in pregnant women and infants.
• GBS serotype acquisition and loss is a common occurrence during colonisation.
• RAPD PCR can be used as a screening tool to identify genetically distinct strains.
• Assay may be useful to complement serotyping before sending GBS isolates for further characterisations.

## 1 Introduction

Group B *Streptococcus* (GBS, *S. agalactiae*) is a gram-positive opportunistic pathogen that causes sepsis, meningitis, and pneumonia in neonates younger than three months. GBS contributes to the gastrointestinal and genitourinary tract microbiota in over 18% of pregnant women worldwide (Russell et al., 2017) and due to maternal colonisation, vertical transmission is the greatest risk factor for disease in neonates during their first seven days of life (Shabayek and Spellerberg, 2018).

There are currently ten serotypes, Ia, Ib, II-IX, characterised by the polysaccharide capsule. Serotype III accounts for 60% of all infant invasive disease cases reported globally (Bianchi-Jassir et al., 2020; Madrid et al., 2017) and 97% of all colonising serotypes are attributable to serotypes Ia, Ib, II-V. Monitoring of both colonising and disease-causing serotypes is an important strategy to inform vaccine development, and post-licensure to monitor for serotype replacement and capsular switching in response to selective pressure following vaccination. Often in epidemiological studies of colonisation only one colony is selected to represent the participant's serotype. Due to the lack of multiple colony testing, colonisation of multiple serotypes can be overlooked, providing an incomplete picture of maternal colonisation and subsequent infant risk of disease.

There have been reports from the USA (Khatami et al., 2019), Spain (Pérez-Ruiz et al., 2004) and France (Beauruelle et al., 2020) demonstrating that multiple GBS serotypes can be present in carriage samples from a small number of women, but information on how commonly this occurs is lacking from other countries. Multiple serotype colonisation increases the risk of serotype replacement in the context of any proposed capsular polysaccharide-based vaccine as seen in *Streptococcus pneumoniae* after the introduction of the PCV7 vaccine which led to a shift in colonisation of non-vaccine serotypes (Huang et al., 2005).

Molecular techniques such as multilocus sequence typing (MLST), which is sequencing of the seven house-keeping genes (Jones et al., 2003), have been used to characterise GBS strains. Other genetic differentiation techniques such as random amplification of polymorphic DNA (RAPD) PCR has been applied to GBS strains to infer transmission by characterising the strains based on their RAPD fingerprint patterns (Brandolini et al., 2014), and this technique is a cheaper and less complex alternative to MLST and whole genome sequencing (WGS). RAPD PCR is not able to specify the strain genotype as there is not a reference for the fingerprint patterns generated, however, it can inform of presence of different genotypes. We sought to investigate the presence of multiple strain co-colonisation of GBS in pregnant women and infants from The Gambia using RAPD PCR as a screening tool to detect genotypically diverse GBS strains, that were then confirmed by WGS.

## 2 Materials and methods

### 2.1 Patient samples

A sub-set of 96 GBS-positive swabs and breastmilk samples, previously collected from the cohort of Gambian women and their infants in 2014 (Le Doare et al., 2016), were used in this study. In total, swabs, and breastmilk samples from 21 mothers and 23 infants from diverse anatomical sites: maternal rectovaginal (*n* = 21), breastmilk (*n* = 9), infant rectal (*n* = 34) and infant nasopharyngeal (*n* = 32) sites, were used. All swabs were stored in skim-milk tryptone glucose glycerol (STGG) transport media and breastmilk samples were kept frozen at -80 °C.

### 2.2 GBS isolation and multiple colony selection

200 μl of STGG or breastmilk was inoculated into 2 ml of LIM RambaQUICK StrepB (CHROMagar, France) and incubated aerobically for 24 h at 37 °C with 5% CO_2_. 10 μl of bacterial culture was streaked onto CHROMagar (CHROMagar, France) and incubated overnight at 37 °C with 5% CO_2_. Each plate was split into four quadrants with an average of 10 [range = 1–20] putative GBS colonies selected per plate and subjected to species confirmation through MALDI-TOF MS (Bruker, USA) using the direct plating technique (To et al., 2019). Subsequently, identified GBS bacterial isolates were stored in 20% *v*/v glycerol at -80 °C.

### 2.3 DNA extraction

Genomic DNA from GBS bacterial isolates was extracted using the Qiagen DNeasy kit (Qiagen, Germany) following the gram-positive bacteria protocol (Qiagen, 2006) with modifications as follows: GBS was lysed in a lysis buffer containing 20 μl mutanolysin (3000 U/ml), 20 μl lysozyme (100 mg/ml) and 4 μl RNaseA (100 mg/ml) prior to incubation at 37 °C for 2 h. The subsequent steps followed the manufacturers instructions. DNA was quantified using a NanoDrop spectrophotometer (Thermo, USA).

### 2.4 RAPD PCR

Extracted genomic DNA was used to conduct a randomly amplified polymorphic DNA (RAPD) PCR, using primer sequence GBS2 (5’-AGAGGGCACA-3′) (Zhang et al., 2002). In brief, PCR reactions were heated at 95 °C for 15 min, then 35 cycles of denaturing at 94 °C for 1 min, annealing at 42 °C for 1 min, extension at 72 °C for 2 min and a final cycle of extension at 72 °C for 5 min on a Veriti thermal cycler (Thermo, USA). The amplified products were visualised on a 1.5% agarose gel and visualised on a ChemiDocTM MP Imaging System (Bio-Rad, USA). GBS reference strains (Table S1) representing serotypes Ia, Ib and III were used as positive controls. More details in Suppl. Methods.

### 2.5 Whole genome sequencing and genomic analyses

Isolates with a unique RAPD pattern from each swab were selected for whole genome sequencing (WGS). Isolates were sequenced on the Illumina Next-Seq platform (Illumina, USA) with 150 bp paired-end reads. Sequence reads quality was checked using FastQC v0.11.8 (http://www.bioinformatics.babraham.ac.uk/projects/fastqc) and raw reads were trimmed using Trimmomatic v0.36 (Bolger et al., 2014), then assembled using SPAdes v3.11.1 (Bankevich et al., 2012) and checked using QUAST v4.5 (Gurevich et al., 2013) resulting in 167 high quality genome sequences for SNP, MLST and serotype analyses and for 15 sequences only MLST and serotype information could be extrapolated. Species identification was achieved using Kraken2 v2.1.1 (Wood and Salzberg, 2014) and KmerFinder 3.0.2 on Center for Genomic Epidemiology (http://www.genomicepidemiology.org). MLST was assigned using SRST2 v0.2.0 (Inouye et al., 2014) and clonal complexes (CC) were assigned using PHYLOViZ (https://www.phyloviz.net). *In-silico* serotyping was done by iPCRess v2.2 (Slater and Birney, 2005) using GBS multiplex primers (Imperi et al., 2010). Single nucleotide polymorphism (SNP) calling to differentiate between strains was done using Snippy v3 (https://github.com/tseemann/snippy), mapped to a reference sequence *S. agalactiae* SS1 serotype V ST1 (GenBank: CP010867.1).

### 2.6 Statistical analysis

An unpaired Mann-Whitney U *t*-test was used on GraphPad Prism v8 to determine the statistical significance between SNPs from isolates with identical and different RAPD patterns. An one-way ANOVA was used to test the statistical significance of increased RAPD pattern diversity between different anatomical sites. *p* < 0.05 represented statistical significance.

## 3 Results

### 3.1 Validation of RAPD PCR ability to differentiate between genetically distinct GBS strains

We applied RAPD PCR assay on 31 swabs from three healthy pregnant women and five infants (see Suppl. material for more details). Twenty-one swabs had GBS colonies with the same RAPD pattern (example in Fig. S2A), and eight swabs had more than one RAPD pattern per swab (example in Fig. S2B), suggesting co-colonisation of multiple strains. For each unique RAPD pattern present in single swab, we selected three representative colonies, where possible, for WGS.

Amongst the identical RAPD patterns that originated from the same swab, there was an average of 2 SNPs (observed range 0-7SNPs). Conversely, when comparing isolates with different RAPD patterns from the same swab, average of 6491 SNPs (range 520‐11,658 SNPs) was noted (Fig. S2C).

### 3.2 Using RAPD PCR to screen for co-colonisation of multiple GBS strains

From our optimisation results of the RAPD PCR (see Suppl. Methods *Confirming genetic diversity*), we showed that the same RAPD pattern represents the same genotype within the single sample (Fig. S2), and therefore we went forward with selecting only one representative isolate per RAPD pattern for WGS. We utilised RAPD PCR to screen a total of 964 GBS colonies cultured from 96 swabs from 21 mothers and 23 infants. Selecting only one colony for each unique RAPD pattern per swab for WGS, resulted in 182 genome sequences used for detailed analysis. In total, we identified six serotypes (Ia, Ib, II-V), eleven MLST genotypes, and six clonal complexes in the study (Table 1).

**Table 1:** Summary of serotypes, sequence types (ST) and clonal complexes (CC) detected amongst GBS isolates identified from 96 swabs after RAPD PCR assay was applied. The results summarise contribution of each serotype and ST observed (%) to the total number of swabs observed with one, two, three, four, or five RAPD patterns.

Serotype and CC/ST		1 RAPD	2 RAPD	3 RAPD	4 RAPD	5 RAPD
		Pattern	Patterns	Patterns	Patterns	Patterns
		(*n* = 65)	(*n* = 23)	(*n* = 5)	(*n* = 1)	(*n* = 2)
Serotype (no. of swabs)		n (%)	n (%)	n (%)	n (%)	n (%)
Ia (n = 22)		10 (45.5%)	6 (27.3%)	3 (13.6%)	1 (4.5%)	2 (9.1%)
Ib (n = 1)		0	1 (100%)	0	0	0
II (n = 29)		14 (48.3%)	10 (34.5%)	2 (6.9%)	1 (3.4%)	2 (6.9%)
III (n = 8)		4 (50%)	2 (25%)	0	1 (12.5%)	1 (12.5%)
IV (n = 15)		5 (33.3%)	6 (40%)	3 (20%)	0	1 (6.7%)
V (n = 49)		32 (65.3%)	13 (26.5%)	4 (8.2%)	0	0

CC/ST (no. of swabs)						
CC1	ST1 (n = 20)	9 (45%)	9 (45%)	1 (5%)	0	1 (5%)
	ST2 (n = 1)	1 (100%)	0	0	0	0
	ST196 (n = 15)	5 (33.3%)	6 (40%)	3 (20%)	0	1 (6.7%)
	ST1274 (n = 16)	9 (56.3%)	6 (37.5%)	0	1 (6.3%)	0
CC10	ST10 (n = 5)	1 (20%)	3 (60%)	1 (20%)	0	0
CC17	ST17 (n = 6)	3 (50%)	2 (33.3%)	0	1 (16.7%)	0
CC19	ST19 (n = 5)	4 (80%)	0	0	0	1 (20%)
	ST28 (n = 7)	2 (28.6%)	1 (14.3%)	1 (14.3%)	1 (14.3%)	2 (28.6%)
CC23	ST23 (n=22)	10 (45.5%)	6 (27.3%)	3 (13.6%)	1 (4.5%)	2 (9.1%)
CC26	ST26 (n=31)	20 (64.5%)	8 (25.8%)	3 (9.7%)	0	0
	ST1357 (n = 1)	1 (100%)	0	0	0	0

In 65/96 swabs (67.7%) there was presence of a single unique RAPD pattern across all GBS colonies picked from the same swab, indicating single genotype of GBS present. In this set of swabs, overall, five serotypes were found, with the most common being serotype V (*n* = 32/65, 49.2%) followed by serotypes II (*n* = 14/65, 21.5%), Ia (*n* = 10/65, 15.4%), IV (*n* = 5/65, 7.7%) and III (*n* = 4/65, 6.2%). The most common genotypes identified were ST26 (*n* = 20/65, 30.8%), ST23 (*n* = 10/65, 15.4%), ST1274 (*n* = 9/65, 13.8%), ST1 (*n* = 9/65, 13.8%), ST196 (*n* = 5/65, 7.7%), ST19 (*n* = 4/65, 6.2%) ST17 (*n* = 3/65, 4.6%, ST28 (*n* = 2/65, 3.1%), ST2 (*n* = 1/65, 1.5%), ST10 (*n* = 1/65, 1.5%), and ST1357 (*n* = 1/65, 1.5%).

**Fig. 1:**
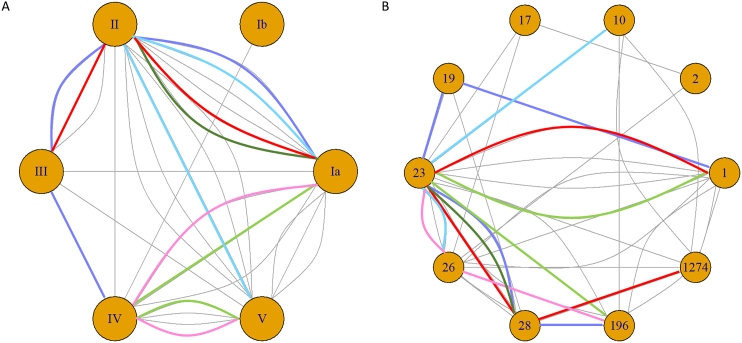
Node plots of (A) serotype and (B) sequence type (ST) highlighting the co-colonisation pairings from 31 swabs that had two or more serotypes/ST present per swab. Six swabs with more than two serotypes/ST are shown by the purple, blue, light green, dark green, pink, and red lines representing the same swab in (A) and (B). The swab represented by the dark green line failed WGS DNA amplification and we were therefore unable to extrapolate ST data for one isolate. Grey lines represent the combinations of only two serotypes/ST during co-colonisation identified from the same swab. (For interpretation of the references to colour in this figure legend, the reader is referred to the web version of this article.)

Of the swabs with more than one unique RAPD pattern (*n* = 31/96, 32.3%), 23 swabs had two different RAPD patterns, five swabs had three, one swab had four and two swabs had five different RAPD patterns (Table 1). Overall, the number of different RAPD patterns matched the number of different serotypes and STs identified within a swab sample. In our dataset, we observed that serotype II, our second most common serotype, was more commonly found in combination with multiple serotypes (Fig. 1A); whilst the most common genotype ST23 was more likely to be found in combination with multiple other MLST types (Fig. 1B).

**Fig. 2:**
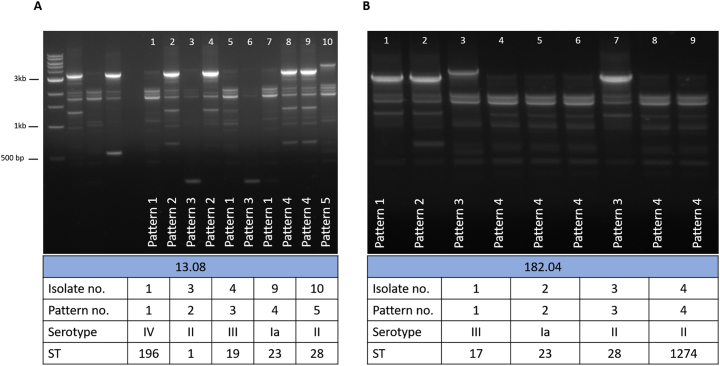
RAPD PCR typing results from two infant rectal swabs where genetic differences were identified through unique RAPD patterns. (A) Five unique RAPD patterns identified five STs and four serotypes amongst ten GBS isolates, lanes 1–5: 1 kb DNA ladder, GBS serotype reference strains NCTC 9993 (serotype Ia), NCTC 8187 (serotype Ib), NCTC 11080 (serotype III), and a negative water control (B) four unique RAPD patterns identified three serotypes and four STs.

**Fig. 3:**
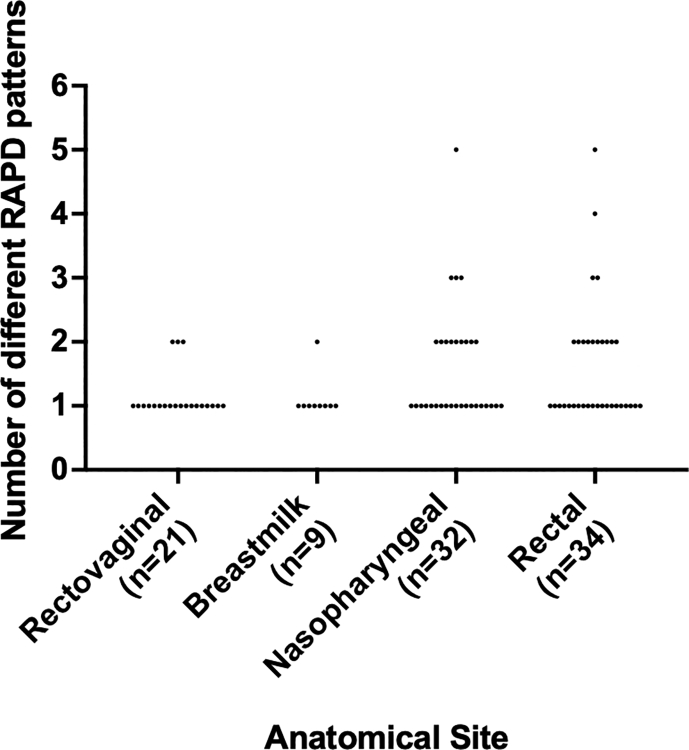
Distribution of the number of different RAPD patterns according to the anatomical origins of the 96 swabs. No statistical significance was observed when comparing between each anatomical site using an one-way ANOVA (*p* > 0.05).

Additionally, we established that RAPD PCR was able to differentiate between colonies with the same serotype and different MLST types (two different RAPD patterns were present) and we have confirmed these different genotypes via WGS. These were from two rectal swabs from two unrelated infants. One swab generated five different RAPD patterns, which after WGS analysis, were found to be four serotypes and five different genotypes: ST1 (serotype II), ST19 (serotype III), ST23 (serotype Ia) ST28 (serotype II) and ST196 (serotype IV) (Fig. 1 purple line, and Fig. 2A). Another swab generated four different RAPD patterns, resulting in three serotypes and four different genotypes: ST17 (serotype III), ST23 (serotype Ia), ST28 (serotype II) and ST1274 (serotype II) (Fig. 1 red line, and Fig. 2A). Overall, we observed that baby rectal swabs yielded two or more RAPD patterns (statistically not significant, Fig. 3). However, the study sample size was too low to determine if the number of unique RAPD patterns truly differed per anatomical site swabbed.

Whole genome sequencing provided additional genotypic information and enabled confirmation of phylogenetic profile in cases with the same serotype showing different RAPD patterns within the sample, later confirmed to be different MLST genotypes. We observed only one outlier case, where one swab from an infant rectum generated two different RAPD patterns suggesting two genetically different strains on the same swab, but these both were serotype II ST28 but with no SNP differences (Fig. S3).

### 3.3 Patterns of GBS co-colonisation detected in mother-infant pairs

**Fig. 4:**
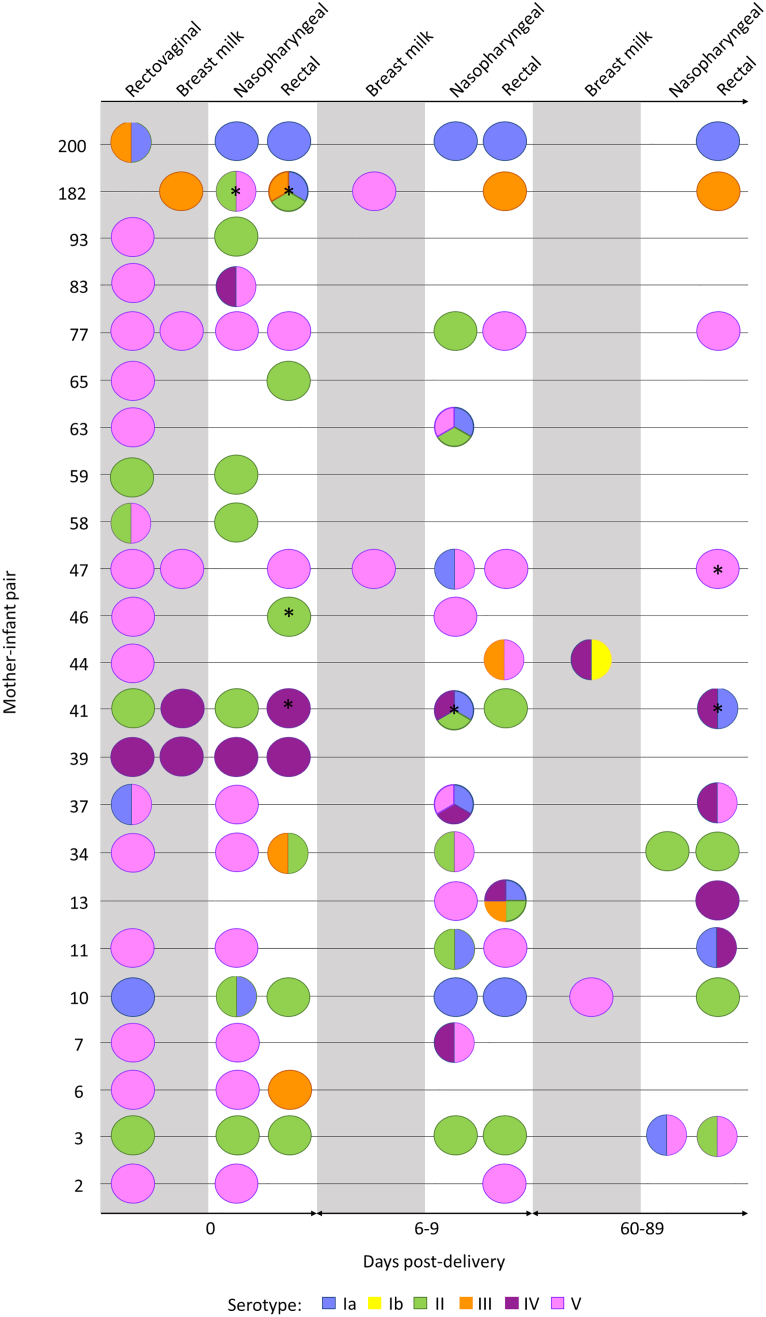
Multiple serotype colonisation of GBS from 96 rectovaginal/breastmilk/nasopharyngeal/rectal swabs originating from 21 mothers and 23 infants where serotype information was extrapolated from WGS. The circles represent the number of different serotypes cultured from each swab. The grey background represents maternal swabs, and the white background represent infant swabs. The swabs were taken at three different timepoints at day 0 (birth), day 6–9 and day 60–89 post-delivery of the infant. * = swab where there was at least one RAPD pattern that was not analysable by WGS. (For interpretation of the references to colour in this figure legend, the reader is referred to the web version of this article.)

Upon examination of serotype colonisation dynamics between mother-infant pairs and infant longitudinal colonisation, we noted several incidences of potential serotype replacement in the infant during their first 60–89 days of life (Fig. 4). We observed that when the mother was co-colonised with multiple serotypes not all of the colonised serotypes were seen to be transmitted to the infant at birth, as seen in mother-infant pair numbers 37, 58 and 200 (Fig. 4). However, if the mother was colonised at the rectovaginal with a serotype different to the breastmilk serotype, both serotypes were seen to be transmitted to the infant at birth but established colonisation at different sites; this was seen in mother-infant pair 41 where the maternal rectovaginal serotype differed to the breastmilk serotype and the two serotypes were found in separate niches in the infant at birth (Fig. 4). However, inference from this study is limited by the low number of strains examined per patient in comparison to colonising bacterial population.

In participants colonised with three serotypes detected from one swab, it was uncommon to see all three serotypes retained during longitudinal colonisation, as seen infants 13 and 182 (Fig. 4). Infant 13 was colonised with five different serotypes (Ia, II, III, IV and V) during day 6–9, but retained only serotype IV at day 60–89 (Fig. 4). Similarly, infant 182 was colonised with four serotypes at birth (Ia, II, III and V) but only serotype III was retained. Overall, from all the mother-infant pairs examined longitudinally during the first 89 days after birth, a mixed population of strains was detected in 16 infants that was a different serotype to the mother (Fig. 4). This supports transient population changes and dominance with acquisition and/or replacement of specific strains in patients over time with an evolving and shifting population.

## 4 Discussion

Applying RAPD PCR to clinically diverse samples, we found that the number of different RAPD patterns not only mirrored the same number of different serotypes but also was able to show differentiation between strains with the same serotype (different genotype). This highlights the need to consider multiple serotype-genotype colonisation of GBS and what implications this has for GBS screening, treatment strategies and, importantly, vaccine design and protective immunity studies.

Multiple serotype carriage of GBS has been reported previously with rates ranging from 6.6% (Khatami et al., 2019) to 21.6% (Ferrieri et al., 2004). We saw a higher proportion of 32.3% of co-colonisation present, represented by more than one RAPD pattern, in any one swab in our cohort. Identifying multiple serotype co-colonisation is important for epidemiological surveillance to ensure less abundant serotypes are not missed that could be responsible for serotype replacement. We observed that mothers and infants can carry multiple serotypes, but these serotypes can be transiently present with acquisition and loss of serotypes being a common occurrence during the first 60–89 days of life of the infant. Extrapolation of data from a limited number of colonies taken from single swabs over time from individual cases may not enable accurate representation of the phylogenetic profile of the entire colonising population of bacteria in a patient. It is likely the detections of multiple strains within patients in this study are an underestimate of what truly is found in nature in differing anatomical sites. GBS is also an important pathogen in the elderly and further studies are required to ascertain differences in the bacterial population/bacteriome and dynamics within patients from birth through to end of life for this pathogen of importance for patients of all ages.

Genomic profiling provides a higher discriminatory power to better understand colonisation dynamics and the potential for multiple strains amongst and between serotypes that could lead to serotype replacement. Molecular methods such as PFGE digestion fingerprinting have been widely used to detect genetic diversity within an individual (Pillai et al., 2009). Another typing method by CRISPR has been employed to understand the heterogenicity of GBS colonisation (Beauruelle et al., 2017), and more recently, a nested qPCR assay was developed to identify serotype co-colonisation (Khatami et al., 2019) and high-throughput characterisation of multiple serotype and strain co-colonisation has been demonstrated by using microarrays for *Streptococcus pneumoniae* from a single swab (Silva et al., 2006; Turner et al., 2011). The latter two methods offer the convenience of capturing the diversity of strains from sweeping the whole agar plate, whereas with PFGE, CRISPR and RAPD PCR require multiple single colonies to be selected and processed and therefore is more cumbersome. In comparison to qPCR and microarray, RAPD PCR provides greater flexibility to perform downstream investigations with the single colonies saved, is rapid and does not require expertise in genomic sequence analysis compared to PFGE and CRISPR. There are limitations to RAPD PCR in that there is not a defined pattern for a specific genotype for GBS and the impact of large recombination events within specific strains may result in different patterns. Nonetheless, we have shown that RAPD PCR is a useful assay to complement serotyping to screen for multiple genetically different strains. Due to the ambiguity of the RAPD PCR fingerprint patterns in some cases, additional genomic discrimination is required. An additional benefit of RAPD is its use as a screening tool for large numbers of isolates, to confirm presence of multiple strain prior to more costly and time-consuming typing systems such as MLST or WGS which could suit a middle-low-income country.

In summary, we show that characterising GBS by RAPD PCR can be a rapid method to screen for presence of multiple strain co-colonisation and can differentiate between different genotypes of the strains with the same serotype. We report that serotype acquisition and loss is a common occurrence with the detection of transient strain populations with acquisition and/or replacement of specific strains in patients over time, as part of an evolving and shifting population. Additional investigations into maternal transmission of GBS to infants and the population dynamics of GBS colonisation and strain dominance and specific tissue tropism are required. The implications of these findings will be important as the current GBS vaccine candidates move towards licensure. Greater understanding of the colonising population dynamics over time in patients is required and will influence studies of protective immunity and strain replacement post-vaccination.

## Data availability

Genomic sequences generated as part of this study are available on the European Nucleotide Archive under project accession number PRJEB46377, with sample accession numbers ERS7160066 to ERS7160247. For metadata, see Table S2.

## Ethics approval

The study was approved by the joint Gambian Government/Medical Research Council Research Ethics Committee, SCC 1350 v4.

## Funding

This work was supported by 10.13039/100010269The Wellcome Trust [104482/Z/14/Z], 10.13039/100005627The Thrasher Research Fund [15055], 10.13039/100000865The Bill & Melinda Gates Foundation [OPP1153630] and 10.13039/501100000833The Rosetrees Trust & The Stoneygate Trust [M683]. The funders had no role in study design, data collection, analysis, decision to publish, or preparation of the manuscript.

## Declaration of Competing Interest

The authors declare that they have no known competing financial interests or personal relationships that could have appeared to influence the work reported in this paper

## Appendix A Supplementary data

Supplementary material Image 1
