# Supplementary material for: RAPD PCR detects co-colonisation of multiple group B streptococcus genotypes: A practical molecular technique for screening multiple colonies
Source: J Microbiol Methods. 2021 Nov;190:106322. doi: 10.1016/j.mimet.2021.106322 (PMC9395992; doi:10.1016/j.mimet.2021.106322)
Supplement: Supplementary file 1 — Supplementary material [file mmc1.docx]

**Supplementary Materials**

**Supplementary Methods**

**RAPD PCR primer selection**

To identify a RAPD PCR primer that offers a range of different DNA fingerprints to differentiate between genotypically unique strains measured by the band patterns, we screened three RAPD primers OPB17 (Martinez et al. 2000), GBS2 (Zhang, Kotiw, and Daggard 2002) and 1254 (Duarte et al. 2004) on NCTC serotype strains representing serotypes Ia, Ib, II-VIII (Table S1). In-house laboratory *E. coli* and *S. pneumoniae* strains were used as negative controls to confirm for RAPD primer specificity for GBS. Each of the three primers gave a fingerprint pattern for each serotype strain tested (Figure S1), however, primer GBS2 gave a clearer distinction between the different serotypes (Figure S1B) measured by the number of bands and band intensity for each serotype. There was little distinction between serotypes Ib, II, III, IV, VI-VIII when applying primer OPB17 (Figure S1A), and primer 1254 gave similar fingerprint patterns for serotypes Ib and II, and serotypes IV-VIII (Figure S1C). GBS2 was used to screen isolates from clinical samples to investigate for GBS within-host genomic diversity.

**GBS colony selection**

To determine whether primer GBS2 could inform us of genetic relatedness whereby the same fingerprint represents strains that are genetically similar, and a different band pattern represents a different strain, we applied the RAPD PCR assay on 31 swabs from three healthy pregnant women and five infants. From each of the 31 swabs, up to 20 colonies per plate, whenever possible, were picked, resulting in 337 GBS colonies for which we screened using RAPD PCR. One swab had only a single culturable colony. Twenty-one out of 30 swabs that had more than one GBS colony showed the same RAPD pattern and exhibited no differences between the bands for all isolates, an example show in Figure S2A. In the remaining eight swabs, there was presence of more than one RAPD pattern per swab, an example shown in Figure S2B, suggesting co-colonisation of multiple strains.

**Confirming genetic diversity**

For each unique RAPD pattern present in a swab, we selected three representative isolates, where possible, for whole genome sequencing to determine whether isolates with identical RAPD patterns are near-identical in their core genomes characterised by the number of SNP differences. Among the identical RAPD patterns that originated from the same swab, there was an average of 2 SNPs observed (range = 0-7SNPs), an example shown in Figure S2A. Conversely, when comparing isolates with different RAPD patterns from the same swab, average 6,491 SNPs (range 520-11,658 SNPs), example shown in Figure S2B, with one outlier where two different RAPD patterns from the same swab were genetically identical (Figure S3). A total of 45 comparisons among 56 isolates representing the same RAPD pattern were compared and 37 comparisons among 19 isolates with different RAPD pattern (Figure S2C).

**Supplementary Tables and Figures**

**Table S1**: GBS reference strains used in the RAPD PCR assay. Strains NCTC 993 (serotype Ia), NCTC 8187 (serotype Ib) and NCTC 11080 (serotype III) showed most distinct RAPD fragmentation and were selected as positive controls for RAPD PCR assay in this study.

| **Strain ID (NCTC)** | **Serotype** |
| --- | --- |
| 9993 | Ia |
| 8187 | Ib |
| 11079 | II |
| 11080 | III |
| 11930 | IV |
| 13946 | V |
| 13947 | VI |
| 13949 | VII |
| 13948 | VIII |


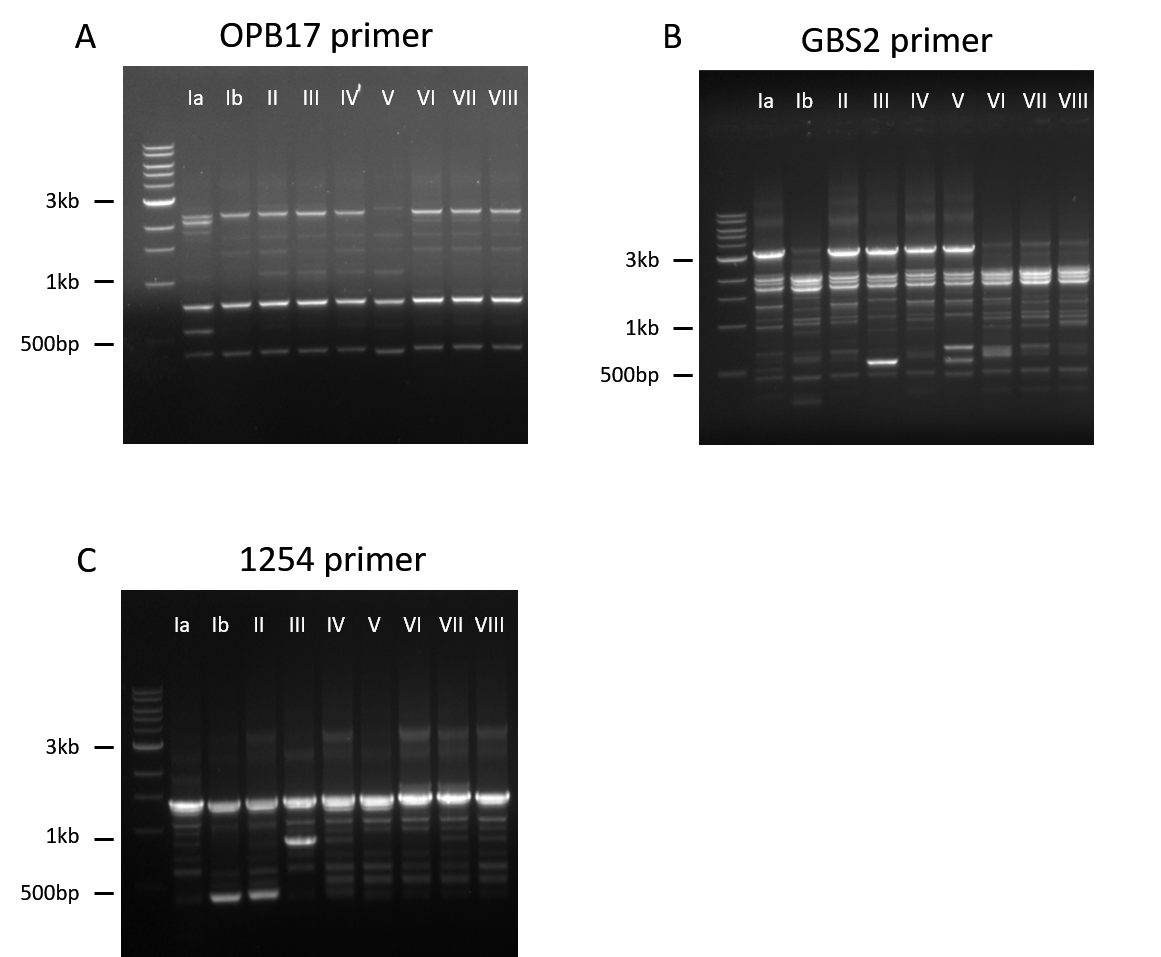


**Figure S1:** RAPD PCR primer testing was carried out on nine GBS serotype reference strains NCTC 9993 (serotype Ia), NCTC 8187 (serotype Ib), NCTC 11079 (serotype II) NCTC 11080 (serotype III), NCTC 11930 (serotype IV), NCTC 13946 (serotype V), NCTC 13947 (serotype VI), NCTC 13949 (serotype VII), NCTC 13948 (serotype VIII on primers: (A) OPB17; (B) GBS2 and (C) 1254. Water was used as a negative control.


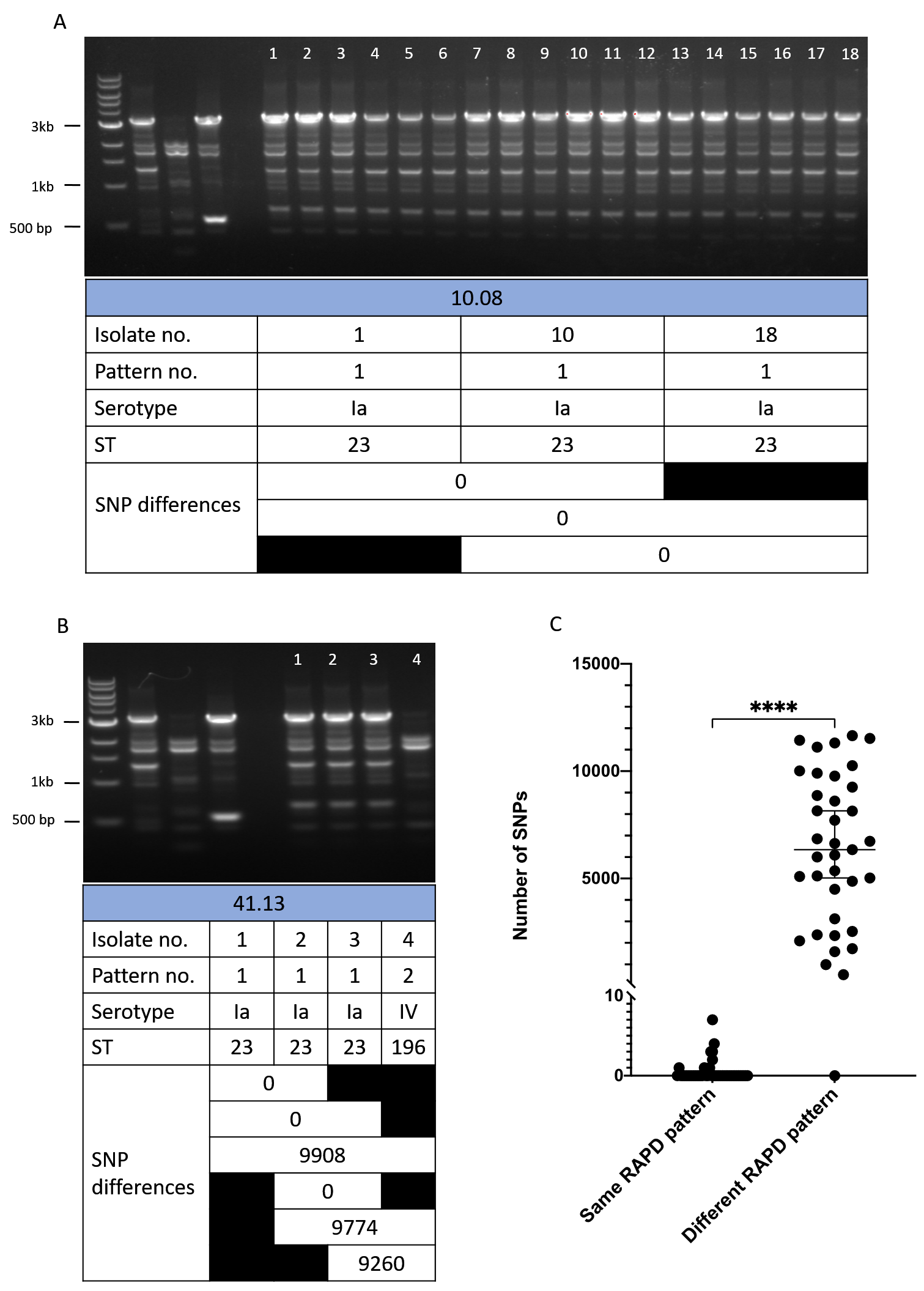


**Figure S2:** RAPD PCR with primer GBS2 can differentiate between genotypically different GBS strains. RAPD typing on two rectal swabs where lanes 1-4 are: 1kb ladder, GBS serotype reference strains NCTC 9993 (serotype Ia), NCTC 8187 (serotype Ib) and NTCT 11080 (serotype III), respectively, and lane 5 a negative water control. (A) lanes 6-23: 18 GBS isolates showing identical RAPD fingerprint patterns with no SNP differences observed between isolate number 1, 10 and 18 (all pattern 1); (B) lanes 6-9: four GBS isolates with two unique RAPD fingerprint patterns where no SNP differences were observed between isolates 1, 2, and 3 (all the same RAPD pattern 1) but at least 9,260 SNPs detected when different RAPD patterns were compared represented by isolate 4; (C) number of SNP differences between GBS isolates with the same RAPD pattern (45 comparisons among 56 genome sequences) ranged from 0–7 SNPs, whilst different RAPD patterns (37 comparisons among 19 genome sequences) ranged from 520–11,658 SNPs with one outlier case of 0 SNPs. **** = p <0.0001 from an unpaired Mann-Whitney U test.


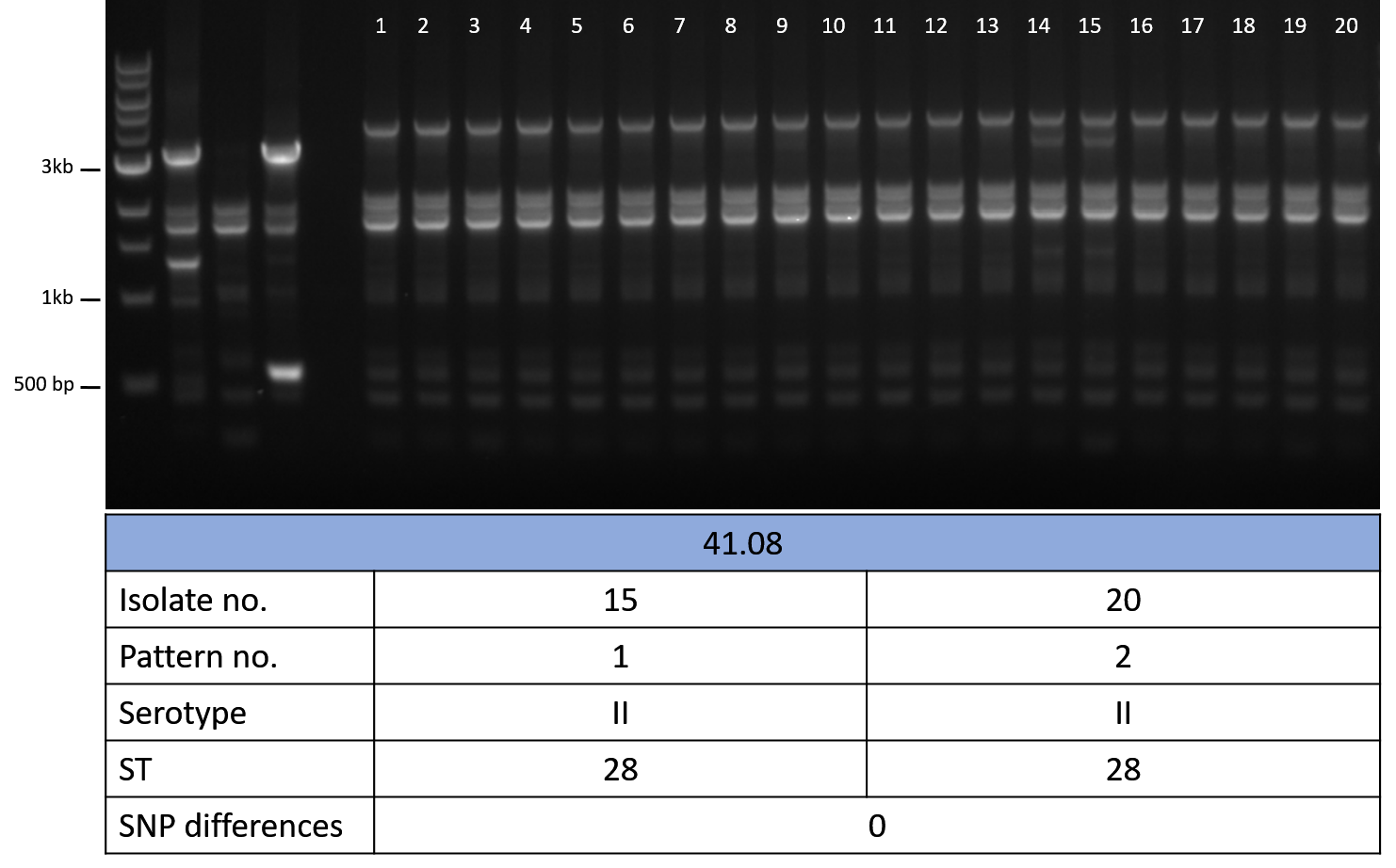


**Figure S3**: Outlier case of RAPD PCR typing on an infant rectal swab on 20 isolates. Two unique RAPD patterns were generated by isolate 14/15 (pattern 1) and 20 (pattern 2) but upon SNP comparison, isolate 15 and 20 were identical. Lanes 2-5: GBS serotype reference strains NCTC 9993 (serotype Ia), NCTC 8187 (serotype Ib), NCTC 11080 (serotype III), and a negative water control.

**Table S2:** Accession numbers, clonal complex (CC), sequence types (ST) and serotype metadata for all genomic sequences

| **Accession Number** | **Sample ID** | **Used for SNP analysis or MLST/serotype** | **Serotype** | **CC** | **ST** |
| --- | --- | --- | --- | --- | --- |
| ERS7160066 | 1001_2 | SNP | Ia | 23 | 23 |
| ERS7160067 | 1003_1 | SNP | II | 1 | 1274 |
| ERS7160068 | 1003_2 | SNP | II | 1 | 1274 |
| ERS7160069 | 1003_3 | SNP | Ia | 23 | 23 |
| ERS7160070 | 1003_4 | SNP | Ia | 23 | 23 |
| ERS7160071 | 1004_10 | SNP | Ia | 1 | 2 |
| ERS7160234 | 1004_5 | MLST/sero | Ia | 1 | 2 |
| ERS7160072 | 1007_1 | SNP | Ia | 23 | 23 |
| ERS7160073 | 1007_10 | SNP | Ia | 23 | 23 |
| ERS7160074 | 1008_1 | SNP | Ia | 23 | 23 |
| ERS7160075 | 1008_10 | SNP | Ia | 23 | 23 |
| ERS7160076 | 1008_18 | SNP | Ia | 23 | 23 |
| ERS7160077 | 1013_1 | SNP | II | 23 | 23 |
| ERS7160078 | 1013_5 | SNP | II | 23 | 23 |
| ERS7160079 | 1014_10 | SNP | V | 26 | 26 |
| ERS7160080 | 1101_1 | SNP | V | 26 | 26 |
| ERS7160081 | 1103_1 | SNP | V | 26 | 26 |
| ERS7160082 | 1107_1 | SNP | Ia | 23 | 23 |
| ERS7160083 | 1107_3 | SNP | II | 1 | 1274 |
| ERS7160084 | 1108_1 | SNP | V | 26 | 26 |
| ERS7160085 | 1113_1 | SNP | IV | 1 | 196 |
| ERS7160239 | 1113_2 | MLST/sero | V | 26 | 26 |
| ERS7160086 | 1113_7 | SNP | Ia | 23 | 23 |
| ERS7160088 | 1307_10 | SNP | V | 1 | 1 |
| ERS7160089 | 1307_20 | SNP | V | 1 | 1 |
| ERS7160090 | 1308_1 | SNP | IV | 1 | 196 |
| ERS7160091 | 1308_10 | SNP | II | 19 | 28 |
| ERS7160092 | 1308_3 | SNP | II | 1 | 1 |
| ERS7160093 | 1308_4 | SNP | III | 19 | 19 |
| ERS7160094 | 1308_9 | SNP | Ia | 23 | 23 |
| ERS7160095 | 1313_1 | SNP | IV | 1 | 196 |
| ERS7160096 | 1313_2 | SNP | IV | 1 | 196 |
| ERS7160097 | 1313_5 | SNP | IV | 1 | 196 |
| ERS7160098 | 18203_1 | SNP | V | 26 | 26 |
| ERS7160099 | 18203_4 | SNP | II | 19 | 28 |
| ERS7160100 | 18204_1 | SNP | III | 17 | 17 |
| ERS7160101 | 18204_2 | SNP | Ia | 23 | 23 |
| ERS7160102 | 18204_3 | SNP | II | 19 | 28 |
| ERS7160103 | 18204_4 | SNP | II | 1 | 1274 |
| ERS7160104 | 18204_5 | SNP | II | 1 | 1274 |
| ERS7160105 | 18204_6 | SNP | II | 1 | 1274 |
| ERS7160106 | 18206_10 | SNP | III | 19 | 19 |
| ERS7160107 | 18206_5 | SNP | III | 19 | 19 |
| ERS7160108 | 18208_1 | SNP | III | 17 | 17 |
| ERS7160109 | 18208_10 | SNP | III | 17 | 17 |
| ERS7160110 | 18209_1 | SNP | V | 26 | 26 |
| ERS7160111 | 18209_10 | SNP | V | 26 | 26 |
| ERS7160112 | 18209_5 | SNP | V | 26 | 26 |
| ERS7160113 | 18213_10 | SNP | III | 17 | 17 |
| ERS7160114 | 18213_5 | SNP | III | 17 | 17 |
| ERS7160115 | 20001_1 | SNP | Ia | 23 | 23 |
| ERS7160116 | 20001_5 | SNP | Ia | 23 | 23 |
| ERS7160117 | 20003_1 | SNP | Ia | 23 | 23 |
| ERS7160118 | 20003_10 | SNP | Ia | 23 | 23 |
| ERS7160119 | 20003_20 | SNP | Ia | 23 | 23 |
| ERS7160120 | 20004_1 | SNP | Ia | 23 | 23 |
| ERS7160121 | 20004_10 | SNP | Ia | 23 | 23 |
| ERS7160235 | 20004_20 | MLST/sero | Ia | 23 | 23 |
| ERS7160122 | 20007_1 | SNP | Ia | 23 | 23 |
| ERS7160123 | 20007_5 | SNP | Ia | 23 | 23 |
| ERS7160124 | 20007_9 | SNP | Ia | 23 | 23 |
| ERS7160125 | 20008_1 | SNP | Ia | 23 | 23 |
| ERS7160126 | 20008_10 | SNP | Ia | 23 | 23 |
| ERS7160127 | 20008_5 | SNP | Ia | 23 | 23 |
| ERS7160236 | 20013_5 | MLST/sero | Ia | 23 | 23 |
| ERS7160128 | 20013_9 | SNP | Ia | 23 | 23 |
| ERS7160129 | 201_1 | SNP | V | 26 | 26 |
| ERS7160130 | 203_1 | SNP | V | 1 | 1 |
| ERS7160131 | 203_2 | SNP | V | 1 | 1 |
| ERS7160132 | 208_1 | SNP | V | 26 | 26 |
| ERS7160133 | 208_8 | SNP | V | 26 | 26 |
| ERS7160134 | 301_1 | SNP | II | 1 | 1274 |
| ERS7160135 | 303_1 | SNP | II | 1 | 1274 |
| ERS7160136 | 304_1 | SNP | II | 1 | 1274 |
| ERS7160137 | 307_1 | SNP | II | 1 | 1274 |
| ERS7160138 | 307_6 | SNP | II | 1 | 1274 |
| ERS7160139 | 308_1 | SNP | II | 1 | 1274 |
| ERS7160140 | 312_1 | SNP | V | 26 | 26 |
| ERS7160141 | 312_3 | SNP | Ia | 23 | 23 |
| ERS7160142 | 313_1 | SNP | II | 1 | 2 |
| ERS7160143 | 313_11 | SNP | V | 26 | 26 |
| ERS7160144 | 3401_1 | SNP | V | 19 | 19 |
| ERS7160145 | 3403_1 | SNP | V | 19 | 19 |
| ERS7160146 | 3404_1 | SNP | II | 1 | 2 |
| ERS7160147 | 3404_11 | SNP | III | 17 | 17 |
| ERS7160148 | 3407_1 | SNP | II | 1 | 1274 |
| ERS7160149 | 3407_2 | SNP | V | 1 | 1 |
| ERS7160150 | 3407_9 | SNP | V | 1 | 1 |
| ERS7160151 | 3412_1 | SNP | II | 1 | 1274 |
| ERS7160152 | 3412_2 | SNP | II | 10 | 10 |
| ERS7160153 | 3413_1 | SNP | II | 1 | 1 |
| ERS7160154 | 3701_1 | SNP | V | 1 | 1 |
| ERS7160155 | 3701_7 | SNP | Ia | 23 | 23 |
| ERS7160156 | 3703_1 | SNP | V | 1 | 1 |
| ERS7160157 | 3707_1 | SNP | Ia | 23 | 23 |
| ERS7160158 | 3707_2 | SNP | V | 1 | 1 |
| ERS7160159 | 3707_4 | SNP | IV | 1 | 196 |
| ERS7160160 | 3713_1 | SNP | IV | 1 | 196 |
| ERS7160161 | 3713_5 | SNP | V | 26 | 26 |
| ERS7160162 | 3713_9 | SNP | IV | 1 | 196 |
| ERS7160163 | 3901_1 | SNP | IV | 1 | 196 |
| ERS7160164 | 3903_1 | SNP | IV | 1 | 196 |
| ERS7160165 | 3904_1 | SNP | IV | 1 | 196 |
| ERS7160166 | 3904_3 | SNP | IV | 1 | 196 |
| ERS7160167 | 3905_1 | SNP | IV | 1 | 196 |
| ERS7160168 | 4101_10 | SNP | II | 19 | 28 |
| ERS7160169 | 4101_12 | SNP | II | 19 | 28 |
| ERS7160170 | 4103_1 | SNP | II | 19 | 19 |
| ERS7160171 | 4103_10 | SNP | II | 19 | 28 |
| ERS7160172 | 4103_18 | SNP | II | 19 | 28 |
| ERS7160237 | 4103_20 | MLST/sero | II | 19 | 19 |
| ERS7160173 | 4104_1 | SNP | IV | 1 | 196 |
| ERS7160174 | 4104_5 | SNP | IV | 1 | 196 |
| ERS7160175 | 4104_8 | SNP | IV | 1 | 196 |
| ERS7160238 | 4105_1 | MLST/sero | IV | 1 | 196 |
| ERS7160176 | 4107_2 | SNP | II | 19 | 28 |
| ERS7160177 | 4107_3 | SNP | Ia | 23 | 23 |
| ERS7160178 | 4107_5 | SNP | Ia | 23 | 23 |
| ERS7160179 | 4107_6 | SNP | II | 19 | 28 |
| ERS7160180 | 4107_7 | SNP | II | 19 | 28 |
| ERS7160087 | 4107_8 | MLST/sero | II | 19 | 28 |
| ERS7160181 | 4108_15 | SNP | II | 19 | 28 |
| ERS7160182 | 4108_20 | SNP | II | 19 | 28 |
| ERS7160183 | 4113_1 | SNP | Ia | 23 | 23 |
| ERS7160184 | 4113_2 | SNP | Ia | 23 | 23 |
| ERS7160185 | 4113_3 | SNP | Ia | 23 | 23 |
| ERS7160186 | 4113_4 | SNP | IV | 1 | 196 |
| ERS7160187 | 4401_1 | SNP | V | 26 | 26 |
| ERS7160188 | 4408_1 | SNP | III | 17 | 17 |
| ERS7160240 | 4408_2 | MLST/sero | V | 26 | 26 |
| ERS7160189 | 4409_1 | SNP | IV | 1 | 196 |
| ERS7160190 | 4409_9 | SNP | Ib | 10 | 10 |
| ERS7160191 | 4601_1 | SNP | V | 26 | 26 |
| ERS7160241 | 4604_9 | MLST/sero | II | 1 | 1274 |
| ERS7160192 | 4607_1 | SNP | V | 1 | 1 |
| ERS7160193 | 4607_3 | SNP | V | 1 | 1 |
| ERS7160194 | 4701_1 | SNP | V | 26 | 26 |
| ERS7160242 | 4704_1 | MLST/sero | V | 26 | 26 |
| ERS7160195 | 4705_1 | SNP | V | 26 | 26 |
| ERS7160196 | 4707_1 | SNP | Ia | 23 | 23 |
| ERS7160197 | 4707_4 | SNP | V | 1 | 1 |
| ERS7160198 | 4708_1 | SNP | V | 26 | 26 |
| ERS7160199 | 4709_1 | SNP | V | 26 | 26 |
| ERS7160200 | 4713_1 | SNP | V | 26 | 26 |
| ERS7160243 | 5801_4 | MLST/sero | II | 1 | 1274 |
| ERS7160244 | 5801_5 | MLST/sero | V | 26 | 26 |
| ERS7160201 | 5803_1 | SNP | II | 1 | 1274 |
| ERS7160202 | 5901_1 | SNP | II | 1 | 1274 |
| ERS7160203 | 5903_1 | SNP | II | 1 | 1274 |
| ERS7160204 | 601_1 | SNP | V | 26 | 26 |
| ERS7160205 | 603_1 | SNP | V | 26 | 26 |
| ERS7160206 | 604_1 | SNP | III | 17 | 17 |
| ERS7160207 | 604_8 | SNP | III | 17 | 17 |
| ERS7160208 | 6301_1 | SNP | V | 19 | 19 |
| ERS7160209 | 6301_4 | SNP | V | 19 | 19 |
| ERS7160210 | 6307_1 | SNP | II | 10 | 10 |
| ERS7160211 | 6307_10 | SNP | V | 26 | 26 |
| ERS7160212 | 6307_7 | SNP | Ia | 23 | 23 |
| ERS7160213 | 6501_1 | SNP | V | 26 | 26 |
| ERS7160214 | 6501_2 | SNP | V | 26 | 26 |
| ERS7160215 | 6504_1 | SNP | II | 1 | 1274 |
| ERS7160216 | 701_1 | SNP | V | 26 | 26 |
| ERS7160217 | 703_1 | SNP | V | 1 | 1 |
| ERS7160218 | 707_1 | SNP | IV | 1 | 196 |
| ERS7160219 | 707_3 | SNP | V | 1 | 1 |
| ERS7160220 | 7701_1 | SNP | V | 1 | 1 |
| ERS7160221 | 7701_4 | SNP | V | 26 | 26 |
| ERS7160222 | 7703_1 | SNP | V | 1 | 1 |
| ERS7160223 | 7704_1 | SNP | V | 1 | 1 |
| ERS7160224 | 7704_3 | SNP | V | 26 | 26 |
| ERS7160245 | 7705_5 | MLST/sero | V | 26 | 26 |
| ERS7160225 | 7707_4 | SNP | II | 10 | 10 |
| ERS7160246 | 7708_1 | MLST/sero | V | 26 | 26 |
| ERS7160247 | 7708_2 | MLST/sero | V | 1 | 1 |
| ERS7160226 | 7713_1 | SNP | V | 1 | 1 |
| ERS7160227 | 8301_1 | SNP | V | 26 | 26 |
| ERS7160228 | 8303_1 | SNP | IV | 1 | 196 |
| ERS7160229 | 8303_3 | SNP | V | 1 | 1 |
| ERS7160230 | 9301_5 | SNP | V | 26 | 1357 |
| ERS7160231 | 9301_7 | SNP | V | 26 | 1357 |
| ERS7160232 | 9303_1 | SNP | II | 1 | 1274 |
| ERS7160233 | 9303_10 | SNP | II | 10 | 10 |
